# Mitigation Policies and COVID-19–Associated Mortality — 37 European Countries, January 23–June 30, 2020

**DOI:** 10.15585/mmwr.mm7002e4

**Published:** 2021-01-15

**Authors:** James A. Fuller, Avi Hakim, Kerton R. Victory, Kashmira Date, Michael Lynch, Benjamin Dahl, Olga Henao, CDC COVID-19 Response Team

**Affiliations:** 1 CDC COVID-19 Response Team.

As cases and deaths from coronavirus disease 2019 (COVID-19) in Europe rose sharply in late March, most European countries implemented strict mitigation policies, including closure of nonessential businesses and mandatory stay-at-home orders. These policies were largely successful at curbing transmission of SARS-CoV-2, the virus that causes COVID-19 (*1*), but they came with social and economic costs, including increases in unemployment, interrupted education, social isolation, and related psychosocial outcomes (*2*,*3*). A better understanding of when and how these policies were effective is needed. Using data from 37 European countries, the impact of the timing of these mitigation policies on mortality from COVID-19 was evaluated. Linear regression was used to assess the association between policy stringency at an early time point and cumulative mortality per 100,000 persons on June 30. Implementation of policies earlier in the course of the outbreak was associated with lower COVID-19–associated mortality during the subsequent months. An increase by one standard deviation in policy stringency at an early timepoint was associated with 12.5 cumulative fewer deaths per 100,000 on June 30. Countries that implemented stringent policies earlier might have saved several thousand lives relative to those countries that implemented similar policies, but later. Earlier implementation of mitigation policies, even by just a few weeks, might be an important strategy to reduce the number of deaths from COVID-19.

Using data from 37 European countries, the impact of the timing and stringency of early mitigation policies on cumulative mortality from COVID-19 on June 30 was assessed. Countries with >250,000 inhabitants and for which relevant data were available were included. Mortality data were obtained from the World Health Organization (WHO) Coronavirus Disease Dashboard (*4*). Data on mitigation policies were obtained from the CDC COVID-19 International Taskforce global mitigation database accessible through WHO* (*5*) and the University of Oxford’s Coronavirus Government Response Tracker (*6*), specifically the Oxford Stringency Index (OSI) (*6*), which is a composite index based on nine mitigation policies. These include cancellation of public events, school closures, gathering restrictions, workplace closures, border closures, internal movement restrictions, public transport closure, recommendations to stay at home, and stay-at-home orders; mask requirements are not included. The OSI ranges from 0 to 100 and increases over time if more stringent mitigation policies are implemented or decreases if policies are rescinded (Supplementary Figure, https://stacks.cdc.gov/view/cdc/100148); however, this index is also weighted on the strictness of each policy, which can vary among countries (*6*). For each country, the value of the OSI was extracted on the date that the country first reached a defined threshold of daily mortality from COVID-19 (mortality threshold). This report uses a threshold of a daily rate of 0.02 new COVID-19 deaths per 100,000 population (based on a 7-day moving average); several thresholds were explored,^†^ all of which produced similar results. The mortality threshold is used to identify a common epidemiologic point early in the pandemic in each country to align countries by the progression of their epidemic, rather than by calendar date.

Linear regression was used to assess the association between the OSI on the day the country reached the mortality threshold and cumulative mortality per 100,000 at the end of June 2020. June 30, 2020 was chosen because at that time, the rate of new COVID-19 deaths per 100,000 had dropped to relatively low levels for nearly all 37 countries. The regression model controls for several covariates: the calendar date the mortality threshold was reached, because countries affected later might have had more time to prepare and less time before the fixed endpoint of June 30; hospital beds in the country per 1,000 population as a measure of baseline health care capacity; median age of the population, because age is an important risk factor for death from COVID-19; population density, because urbanization might lead to higher rates of contact; and gross domestic product per capita to account for differences in wealth. Controlling for other OSI metrics (e.g., the mean, median, and maximum OSI from January 1 to June 30) was explored, but none had a meaningful effect on the results. The number of lives lost attributable to a lower OSI on the day the country reached the mortality threshold was calculated using the results from the linear regression. For each country whose OSI was <80 when reaching the mortality threshold, a counterfactual scenario was estimated by calculating the expected reduction in mortality had their OSI been 80.^§^

Among 37 European countries, the date the mortality threshold was reached ranged from March 2 (Italy) to April 18 (Ukraine), and the OSI on the date the mortality threshold was reached ranged from 16.7 (United Kingdom) to 100.0 (Serbia) (Table). The most common policies implemented in these countries by the time they reached the mortality threshold were cancellation of public events (35 countries; 95%), followed by school closures (33; 89%), restrictions on gatherings (31; 84%), workplace closures (31; 84%), border closures (27; 73%), restrictions on internal movement (25; 68%), and recommendations to stay at home (14; 38%). Several countries implemented more stringent policies including closure of public transportation (18; 49%) and stay-at-home orders (11; 30%). Countries with more policies in place generally had a higher OSI; however, several countries had a higher index with fewer policies in place. For example, Serbia (index = 100) and Hungary (index = 76.9) had similar types of policies in place, but Serbia had stricter policies such as restrictions on gatherings of ≥10 persons, compared with Hungary, which had restrictions on gatherings of >1,000 persons.

**TABLE:** Mortality threshold date,* stringency index, and COVID-19 mitigation policies implemented, by Oxford Stringency Index (OSI) on date mortality threshold was reached — 37 European countries, March–April, 2020

Country	Date mortality threshold reached	OSI when mortality threshold reached	Cancellation of public events	School closures	Gathering restrictions	Workplace closures	Border closures	Internal movement restrictions	Public transport closure	Recommendations to stay at home	Stay-at-home orders
United Kingdom	Mar 16	16.7	N	N	N	Y	N	N	N	Y	N
Belarus	Apr 08	18.5	Y	Y	N	N	N	N	N	N	N
Luxembourg	Mar 11	22.2	Y	Y	N	N	N	N	N	N	N
Belgium	Mar 13	23.2	Y	N	N	N	N	N	N	N	N
Switzerland	Mar 10	25.0	Y	N	Y	N	N	N	N	N	N
Sweden	Mar 12	27.8	Y	N	Y	N	N	N	N	N	N
France	Mar 13	41.2	Y	Y	Y	Y	N	N	N	N	N
Spain	Mar 10	45.8	Y	Y	Y	Y	Y	Y	N	N	N
Ireland	Mar 24	48.2	Y	Y	Y	Y	N	N	N	N	N
Iceland	Mar 17	50.9	Y	Y	Y	Y	N	N	N	N	N
Cyprus	Mar 22	51.9	Y	Y	N	Y	Y	N	N	N	N
Netherlands	Mar 15	54.6	Y	Y	Y	Y	N	Y	N	Y	N
Norway	Mar 23	63.0	N	Y	Y	Y	Y	Y	Y	N	N
Finland	Mar 26	64.8	Y	Y	Y	N	Y	Y	N	Y	N
Germany	Mar 21	68.1	Y	Y	Y	Y	Y	Y	N	Y	N
Latvia	Apr 10	69.4	Y	Y	Y	Y	Y	N	Y	Y	N
Italy	Mar 02	69.9	Y	Y	Y	Y	Y	Y	N	Y	N
Bulgaria	Apr 01	71.3	Y	Y	Y	Y	Y	Y	N	Y	N
Denmark	Mar 18	72.2	Y	Y	Y	Y	Y	Y	Y	Y	N
Estonia	Mar 27	72.2	Y	Y	Y	Y	Y	Y	N	N	N
Greece	Mar 22	74.1	Y	Y	Y	Y	Y	Y	Y	N	N
Slovakia	Apr 16	75.0	Y	Y	Y	Y	Y	Y	Y	Y	N
Turkey	Mar 28	75.9	Y	Y	N	Y	Y	Y	Y	Y	N
Hungary^†^	Mar 31	76.9	Y	Y	Y	Y	Y	Y	Y	Y	Y
Romania	Mar 27	78.7	Y	Y	Y	Y	Y	Y	Y	N	Y
Slovenia	Mar 23	78.7	Y	Y	Y	Y	Y	N	Y	Y	N
Austria	Mar 20	81.5	Y	Y	Y	Y	Y	Y	Y	N	Y
Lithuania	Mar 23	81.5	Y	Y	Y	Y	Y	Y	Y	Y	N
Poland	Apr 01	81.5	Y	Y	Y	Y	Y	Y	N	N	Y
Czechia	Mar 27	82.4	Y	Y	Y	Y	Y	Y	N	N	Y
Portugal	Mar 21	82.4	Y	Y	Y	Y	Y	Y	Y	N	Y
Albania	Mar 24	84.3	Y	Y	Y	Y	Y	Y	Y	N	Y
Moldova	Mar 31	87.0	Y	Y	Y	Y	Y	Y	Y	N	Y
Ukraine	Apr 18	88.9	Y	Y	Y	Y	Y	Y	Y	Y	N
Bosnia and Herzegovina	Mar 27	89.8	Y	Y	Y	Y	Y	Y	Y	N	Y
Croatia	Mar 27	96.3	Y	Y	Y	Y	Y	Y	Y	N	Y
Serbia	Mar 27	100.0	Y	Y	Y	Y	Y	Y	Y	N	Y
**Total countries**	—	—	**35**	**33**	**31**	**31**	**27**	**25**	**18**	**14**	**11**

**Abbreviations: **COVID-19 = coronavirus disease 2019; N = no; Y = yes.

* The mortality threshold is the first date that each country reached a daily rate of 0.02 new COVID-19 deaths per 100,000 population based on a 7-day moving average of the daily death rate. “Yes” indicates that the policy was implemented before the date mortality threshold was reached, and “No” indicates that the policy had not been implemented. No country rescinded any policy before the mortality threshold was reached. Implementation of more policies in a country could result in a higher OSI; however, this index is also weighted on the strictness of each policy, which can vary among countries. For example, Serbia (index = 100) and Hungary (index = 76.9) had similar types of policies in place, but Serbia had more strict policies such as restrictions on gatherings of ≥10 persons compared with Hungary, which had restrictions on gatherings of >1,000 persons.

^†^ Hungary implemented a stay-at-home order with exceptions for persons who commuted or had extraordinary situations; these persons were still under recommendations (but not requirements) to stay at home.

Cumulative COVID-19–associated mortality on June 30 was lower in countries that had a higher OSI when reaching the mortality threshold (Figure). This association persisted after controlling for the calendar date the mortality threshold was reached, hospital beds per 1,000 population, median age of the population, population density, and gross domestic product per capita. For each 1-unit increase in the OSI when the mortality threshold was reached, the cumulative mortality as of June 30 decreased by 0.55 deaths per 100,000 (95% confidence interval [CI] = −0.82 to −0.27 deaths per 100,000). A 1-unit increase in the OSI standard deviation (22.9 unit increase in the OSI) was associated with a decrease of 12.5 deaths per 100,000.

**FIGURE:**
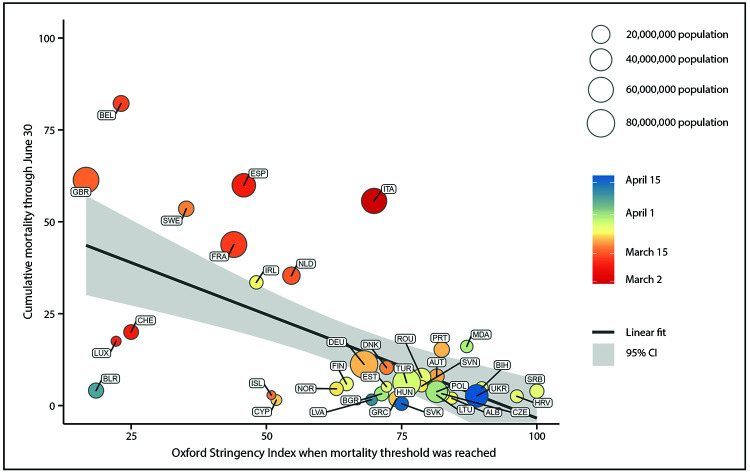
Early policy stringency* and cumulative mortality^†^ from COVID-19 — 37 European countries, January 23–June 30, 2020 **Abbreviations:** ALB = Albania; AUT = Austria; BEL = Belgium; BGR = Bulgaria; BIH = Bosnia and Herzegovina; BLR = Belarus; CHE = Switzerland; CI = confidence interval; COVID-19 = coronavirus disease 2019; CYP = Cyprus; CZE = Czechia; DEU = Germany; DNK = Denmark; ESP = Spain; EST = Estonia; FIN = Finland; FRA = France; GBR = United Kingdom; GRC = Greece; HRV = Croatia; HUN = Hungary; IRL = Ireland; ISL = Iceland; ITA = Italy; LTU = Lithuania; LUX = Luxembourg; LVA = Latvia; MDA = Moldova; NLD = Netherlands; NOR = Norway; POL = Poland; PRT = Portugal; ROU = Romania; SRB = Serbia; SVK = Slovakia; SVN = Slovenia; SWE = Sweden; TUR = Turkey; UKR = Ukraine. * Based on the Oxford Stringency Index (OSI) on the date the country reached the mortality threshold. The OSI is a composite index ranging from 0–100, based on the following nine mitigation policies: 1) cancellation of public events, 2) school closures, 3) gathering restrictions, 4) workplace closures, 5) border closures, 6) internal movement restrictions, 7) public transport closure, 8) stay-at-home recommendations, and 9) stay-at-home orders. The mortality threshold is the first date that each country reached a daily rate of 0.02 new COVID-19 deaths per 100,000 population, based on a 7-day moving average of the daily death rate. The color gradient represents the calendar date that each country reached the mortality threshold. ^†^ Deaths per 100,000 population.

Overall, the OSI was <80 when the mortality threshold was reached in 26 (70%) of 37 countries (Table). On the basis of the regression model, it was determined that if the OSI in each of those countries had been 80 when reaching the mortality threshold, 74,139 fewer deaths would have been expected across those 26 countries. Most of these potentially averted deaths would have been in the United Kingdom (22,776; 31% of all averted deaths), France (13,365; 18%), and Spain (9,346; 13%).

## Discussion

European countries that implemented more stringent mitigation policies by the time they reached an early mortality threshold in spring 2020 tended to report fewer COVID-19–associated deaths through the end of June. Countries that implemented stringent policies earlier might have saved several thousand lives relative to those countries that implemented similar policies, but later. These findings suggest that earlier implementation, even by just a few weeks, might be important to preventing widespread transmission and large numbers of deaths.

Other research has highlighted the importance of the timing of control measures in mitigating the COVID-19 pandemic. One study of the 37 Organization of Economic Cooperation and Development member countries found that implementing school closures and gathering bans 1 week earlier could have reduced mortality by 44% (*7*). A modeling study highlighted a “window of opportunity” for implementing social distancing directives, suggesting that even small delays could lead to much higher incidence rates (*8*). An observational study of 43 U.S. states and 41 countries that implemented stay-at-home orders, found that jurisdictions that delayed those orders experienced more prolonged outbreaks (*9*). Another observational study of U.S. states and other countries found that several nonpharmaceutical interventions, including but not limited to cancelling small gatherings, airport restrictions, and closure of educational institutions, could lead to a larger reduction in transmission if implemented earlier rather than later (*10*).

The findings in this report are subject to at least four limitations. First, some COVID-19 deaths likely went undetected, especially during the early stages of the pandemic. This could impact both the date of reaching the mortality threshold and the cumulative mortality as of June 30. Second, the OSI does not capture all mitigation policies that countries might apply. For example, it does not include requirements for masks, though such requirements in Europe were rare during the early stages of the pandemic. Third, adherence to policies or recommendations was not accounted for and could explain some of the variability in the impact observed. Finally, many interventions were implemented simultaneously, making it difficult to determine which specific policies might have had the most impact.

This report quantifies the impact of earlier implementation of mitigation policies on COVID-19 mortality in Europe during the early stages of the pandemic. Further work should seek to identify optimal timing and duration of mitigation policies, evaluate the role of mask policies in relation to other mitigation policies, and assess which specific interventions are the most effective.

**Summary**

**What is already known about this topic?**

Mitigation policies, including closure of nonessential businesses, restrictions on gatherings and movement, and stay-at-home orders, have been critical to controlling the COVID-19 pandemic in many countries, but they come with high social and economic costs.

**What is added by this report?**

European countries that implemented more stringent mitigation policies earlier in their outbreak response tended to report fewer COVID-19 deaths through the end of June 2020. These countries might have saved several thousand lives relative to countries that implemented similar policies, but later.

**What are the implications for public health practice?**

Earlier implementation of stringent mitigation policies, even by just a few weeks, appears to be important to prevent widespread COVID-19 transmission and reduce the number of deaths.

## Footnotes

* Mitigation policies implemented by government authorities during January 1–June 30, 2020 were abstracted from media reports and government and United Nations websites and compiled by WHO. The CDC COVID-19 International Taskforce global mitigation database is a sub-set of the WHO public health and social measures database.

† The following potential mortality thresholds were explored: number of cumulative deaths (all values between one and 50 deaths), number of cumulative deaths per 100,000 population (all values between 0.01 and 0.5 deaths per 100,000), and the number of daily deaths per 100,000 population (all values between 0.001 and 0.05 deaths per 100,000).

§ The expected reduction in mortality was calculated as the product of three values: 1) the difference between the observed OSI when reaching the mortality threshold and 80, 2) the linear regression coefficient (−0.55), and 3) the population size (measured in 100,000 increments to account for the units of the regression coefficient). A value of 80 for the OSI was selected because it was the average maximum OSI values that countries reached before June 30, 2020.
